# Risk Factors for Recurrent Laryngeal Nerve Palsy in Thyroid Surgery: A Single Center Experience of 1147 Procedures with Intermittent Intraoperative Neuromonitoring

**DOI:** 10.3390/jpm14070714

**Published:** 2024-07-02

**Authors:** Navid Tabriz, Selma Muehlbeyer, Dirk Weyhe, Verena Uslar

**Affiliations:** 1 School VI-School of Medicine and Health Sciences, Carl von Ossietzky Universitat Oldenburg, Ammerlander Heerstrasse 114-118, 26129 Oldenburg, Germany; navid.tabriz@pius-hospital.de (N.T.); dirk.weyhe@pius-hospital.de (D.W.); verena.uslar@pius-hospital.de (V.U.); 2 University Hospital for Visceral Surgery, Pius-Hospital Oldenburg, Carl von Ossietzky Universitat Oldenburg, Georgstrasse, 12, 26121 Oldenburg, Germany

**Keywords:** risk factors, recurrent laryngeal nerve paralysis, thyroid surgery, IONM

## Abstract

Background: Recurrent laryngeal nerve (RLN) palsy is one possible complication during thyroid surgery. Intraoperative neuromonitoring and visualization of the nerve during surgery are standard procedures to reduce the risk of RLN palsy. This study aims to investigate new factors for RLN palsy and review ones that are already known in the literature to help surgeons prepare for the procedure. Methods: A retrospective study design was used to analyze the data of 1147 patients from a certified center for thyroid surgery. All patients underwent either total thyroidectomy or hemithyroidectomy from 2016 to 2020. The acquired information was analyzed descriptively. A logistic regression was used to analyze the independent variables of interest with the binary variable RLN palsy (yes/no). For the second aim of this study, a multiple logistic regression was applied to analyze the combined significant known and new risk factors. Results: Surgery indication for Graves’ disease (OR 14.34, *p* < 0.001), thyroid cancer (OR 2.39, *p* = 0.012), and recurrent goiter (OR 5.57, *p* < 0.001) increased the risk for RLN palsy significantly compared to nodular goiter in hemithyroidectomy. The duration of surgery correlated positively with a higher risk for RLN palsy (OR 1.009, *p* = 0.005). For gender, BMI, resection weight, left or right nerve at risk, and surgeon experience, no significant differences were found. Conclusion: Operations for Graves’ disease, thyroid cancer, and recurrent goiter have the highest risk for RLN palsy and surgeons should be alerted. The longer the operation, the higher the risk of RLN palsy. The correlation between surgery method (hemithyroidectomy vs. thyroidectomy) and RLN palsy should be carefully considered due to possible bias.

## 1. Introduction

Thyroid resections are the most common operations on endocrine organs. Around 60,000 thyroid resections were performed in Germany in 2021 [1]. Apart from postoperative hypoparathyroidism following thyroidectomy, laryngeal nerve (RLN) palsy is the most common complication during thyroid surgery, with a transient rate of 3.6% of nerves at risk in Germany [2]. The RLN innervates all of the intrinsic muscles of the larynx except the cricothyroid muscle. Harming the RLN causes a failure of the intrinsic motoric muscles, resulting in a paramedian or median position of the vocal cord, leading to symptoms such as hoarseness, shortness of breath, or the need for intubation in the case of bilateral palsy. Postoperative RLN palsy is defined as transient if the mobility of the vocal cords returns within six months. Permanent RLN palsy was documented in 1.4% of a German cohort [2]. The visual identification of RLN in thyroid surgery is still the standard procedure to avoid intraoperative injury, but finding the nerve can be very challenging due to its possible anatomical variations and some individual cases, i.e., voluminous glandular goiter or redo surgery [3]. Intraoperative neuromonitoring (IONM) can help to avoid RLN injury [4]. However, its superiority compared to the macroscopic representation of RLN alone is an issue of discussion, particularly with regard to the equal RLN paresis rates, irrespective of IONM use [5]. In Germany, the use of IONM during thyroid surgery has increased from 72.3% in 2008 to 90.9% in 2016 and 98,4% in 2018 [2,6].

However, besides the knowledge of the anatomical course of the laryngeal nerve and its variations, some further factors increasing the risk of nerve palsy are already known. It has been reported that operations for thyroid cancer, Graves’ disease, recurrent goiter, and extensive surgery (total thyroidectomy vs. lobectomy vs. subtotal resection) have an increased risk of post-surgery nerve paralysis [7,8]. Moreover, surgeon experience (50 thyroid operations/year) and hospital volume (>100 thyroid operations/year) can also reduce RLN injury [9,10,11].

Preoperative knowledge of potential risk factors for PLN palsy can help surgeons to better prepare for the operation, possibly adapt their surgical strategy, or use additional devices such as intermittent or continuous IONM in certain cases.

The aim of this study is to analyze known and potential new risk factors for RLN palsy in thyroid surgery using retrospective data for 1147 operations of a certified center for thyroid and parathyroid surgery.

## 2. Materials and Methods

### 2.1. Patients and Data

We conducted a monocentric retrospective study of patients with thyroid surgery between January 2016 and December 2020 in a DGAV (Deutsche Gesellschaft für Allgemein- und Viszeralchirurgie) certified center for thyroid and parathyroid surgery. The patient collective was established using a prospectively maintained database. Information that was not included in the original database was retrospectively acquired and added. Gender, age, BMI (body mass index), indication for surgery, type and duration of procedure, nerves at risk, preoperative laboratory values, specimen weight, perioperative complications, and perioperative laryngoscopy findings were collected.

**Figure 1:**
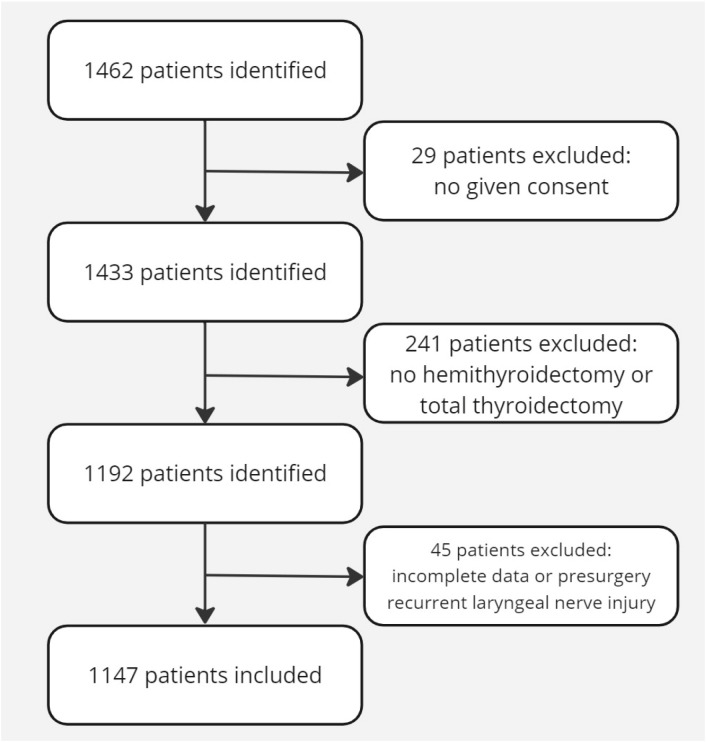
Flowchart of patients identified and included.

In the database, 1462 patients were identified. We excluded 29 patients with no given consent, 241 patients who did not meet the criteria regarding the surgery, 41 patients who had incomplete data, and 4 patients with pre-surgery nerve injury. In the end, a total of 1147 patients, including 102 recurrent laryngeal nerve injuries, were included for analysis (Figure 1).

### 2.2. Surgery and Surgeon Experience

Depending on the indication, patients had either a total thyroidectomy or a hemithyroidectomy. During surgery, the vagus and RLN were presented macroscopically and with intermittent IONM before and after the resection in all cases. Total thyroidectomy had two nerves at risk and hemithyroidectomy had one nerve at risk. In cases of planned thyroidectomy and intraoperative loss of signal (LOS) after the resection of one side, the contralateral side was not addressed, and the operation was ended. For analysis, these patients were assigned to the hemithyroidectomy group.

One of the indications for operation was suspected malignancy due to ultrasonographic criteria and/or fine needle aspiration cytology (Bethesda III-V). In cases of intraoperative histological confirmation of a tumor by frozen section procedure, an ipsilateral central lymph node dissection was performed, but, if LOS occurred, a contra-lateral thyroid exploration was omitted. The vocal cord function was examined on the 1st or 2nd postoperative day by laryngoscopy and if a paresis/paralysis was confirmed, a decision for completion surgery was made on a case-by-case basis regarding whether a two-stage resection was absolutely necessary/indicated according to the tumor formula (>pT1a or pN1a). If a completion surgery was indicated, this was attempted after recovery of the paresis or performed during the index stay as an individualized decision. This patient group was assigned to “hemithyroidectomy in the thyroid cancer group”, and, to avoid the double-counting of these patients, the possible follow-up completion surgery was not counted for analysis.

In cases of the postoperative diagnosis of thyroid cancer and an index hemithyroidectomy, a resection of the contralateral thyroid lobe with centro-cervical lymph node dissection (Kompartment 1 according to Dralle) was performed in all cases in the presence of contralateral thyroid nodules [12,13]. Even in cases of pT1a papillary cancer and contralateral thyroid nodules, a completion surgery with central lymph node dissection was recommended in a patient-centered decision-making process (center’s decision). To avoid the double-counting of patients, the second operation was not listed for evaluation purposes and the index operation was assigned to the “hemithyroidectomy” group.

The re-surgery group consisted of all patients who needed a second surgery during the index inpatient stay, i.e., due to postoperative bleeding, completion surgery for cancer, or Graves´ disease after recovery of RLN paresis. Again, to avoid double-counting for statistical analyses, this group was described descriptively and not included in the evaluation.

All operations were performed by an experienced surgeon or in their presence as an assistant. A surgeon was defined as experienced if more than 50 thyroid surgeries were performed per year.

### 2.3. Recurrent Laryngeal Nerve

Vocal cord function was assessed pre- and postoperatively by fiberoptic laryngoscopy in all cases. Vocal cord paralysis was defined when paramedian positioning compared to the contralateral side was detected. In cases of preoperative evidence of RLN paralysis, the patient was excluded from the study. All operations were performed exclusively using intermittent IONM following the description of Dralle et al. [13]. For those who were diagnosed with postoperative recurrent laryngeal nerve palsy, a logopedic treatment was prescribed and a follow-up laryngoscopy was conducted six months postoperatively.

### 2.4. Statistics

Descriptive analysis was performed for characteristics of patients and variables of interest with SPSS 25 by IBM. Means and standard deviations were used to describe the numeric data. Categorical data were shown as absolute and relative frequencies.

The statistics program JASP 0.12.2 was used for the logistic analysis. The following independent variables were included in a univariable logistic regression with the binary variable RLN injury (yes/no): gender, BMI, resection weight, indication for surgery, surgery duration, surgery method, surgeon experience, and left or right nerve at risk. In addition, the same independent variables were used in an analysis with total thyroidectomies and hemithyroidectomies divided into two separate groups. Furthermore, the significant variables were put together in a multiple logistic regression to analyze a potential increase in the risk.

The significance level was set for all tests with *p* < 0.05. In addition, the odds ratios with corresponding 95% confidence intervals (95% CIs) were calculated for possible risk factors. In the case of multiple testing, a correction was followed according to Benjamini and Hochberg [14].

## 3. Results

### 3.1. Descriptive Analysis

Out of 1147 patients included in this study, approximately 75% (n = 854) were female and 25% (n = 293) were male. The average age was 52 years (range 13–90 years) and 44% (n = 501) of the procedures were total thyroidectomies and 56% (n = 646) were a hemithyroidectomy. There were 1648 nerves at risk (501 bilateral and 646 unilateral). Due to cancer-related completion surgery or complication, i.e., wound infection or hematoma, n = 94 patients needed a second surgery during their hospital stay.

Table 1 shows a comparison of patients‘ characteristics between patients with RLN palsy and patients without RLN palsy split up in hemithyroidectomy and thyroidectomy. A total of 101 patients (6.1%) had recurrent laryngeal nerve paralysis (54 left side, 47 right side). Out of these patients, n = 85 (5.1%) were diagnosed with temporary recurrent laryngeal nerve palsy and n = 16 (1%) patients were diagnosed with permanent paralysis. No patient suffered a bilateral paresis. There were no major differences in gender, age, BMI, surgery duration, duration of stay, or laboratory values between the two groups. The RLN palsy was 6% in total thyroidectomy versus 11% in hemithyroidectomy.

### 3.2. Logistic Regression with Individual Variables

In Graves’ disease, thyroid cancer, and recurrent goiter, a significantly higher risk for RLN palsy was detected compared to benign nodular diseases (Chi^2^ = 8.279, *p* = 0.004, OR = 1.865, 95% CIs = 1.227–2.833). However, if only the Graves’ disease patients were considered, the significance level was not reached (*p* = 0.133). The analyses regarding BMI and gender showed no significant difference in RLN palsy (BMI: Chi^2^ = 0.699, *p* = 0.692 and gender: Chi^2^ = 1.384, *p* = 1.308). Surgery duration (the longer the surgery, the higher the probability for RLN palsy) and surgery method (hemithyroidectomy vs. total thyroidectomy, with hemithyroidectomy presenting a higher risk) were significant factors for RLN palsy (surgery duration: Chi^2^ = 7.753, *p* = 0.005, OR = 1.009, Cis = 1.003–1.015 and surgery method: Chi^2^ = 9.136, *p* = 0.003, OR = 1.939, Cis = 1.244–3.022). Resection weight could not be proven to have a significant impact on having a higher risk for RLN palsy (resection weight: Chi^2^ = 2.957, *p* = 0.086) (see Table 2 below).

### 3.3. Logistic Regression with Individual Variables Separated by Total Thyroidectomies and Hemithyroidectomies

The logistic regression model for only total thyroidectomies showed a significant difference in the risk for RLN palsy for longer surgery duration (see Table 3). Patients with surgery indications for Graves’ disease, thyroid cancer, and recurrent goiter had no significantly higher risk for RLN palsy compared to the other indications. The other variables revealed no significantly higher risk for RLN palsy (see Table 3 below).

The analysis of the hemithyroidectomized patients showed a significant difference in the risk for RLN palsy for Graves’ disease, thyroid cancer, and recurrent goiter compared to the other indications (see Table 4). Furthermore, a significant difference regarding a higher RLN palsy risk for surgery duration and resection weight was found; the longer the surgery and the higher the resection weight, the higher the probability of RLN palsy. A significant difference for a higher RLN palsy risk for the left or right nerve in hemithyroidectomies could not be found.

### 3.4. Multiple Logistic Regression with Significant Variables

The significant variables from the analysis in Table 2 (surgery duration, indication for surgery, and surgery method) were put together to study whether a combination better explained the occurrence of RLN palsy. The variables surgery duration, surgery indication, and surgery method showed a significant difference for a higher risk of RLN palsy (see Table 5). The multiple logistic regression showed a sensitivity of 0.010 and a specificity of 0.999.

## 4. Discussion

This retrospective analysis was set up to determine potential risk factors for RLN palsy in hemi- and total thyroidectomies. Various studies have shown that the indication for surgery has a crucial influence on the outcome in regard to RLN palsy. In particular, surgery for Graves’ disease, thyroid cancer, and recurrent goiter was associated with a higher risk of RLN palsies than surgery for benign nodular goiters [7,8].

This is in line with our results, as patients with thyroid cancer, including central lymph dissection, had a 2.1-fold increased risk of RLN palsy compared to benign nodular goiter, while patients with Graves’ disease had a 1.6-fold increased risk and those with recurrent goiter had a 4.1-fold increased risk. The risk of RLN paresis in thyroid carcinoma depends on the location of the tumor, tumor histology, staging, and the extent of the resection. In a retrospective analysis of thyroid cancer with 400 nerves at risk, an RLN palsy rate of 6.8% was recorded, and the risk of RLN palsy was the highest in the presence of pT4a tumors (OR = 8.5), gross extra-thyroidal extension (OR = 3.5), and tracheo-esophageal groove (TEG) (OR = 2.8) involvement [15].

Lymph node dissection itself and its extension (unilateral central vs. bilateral central vs. lateral) is associated with an increased rate of RLN paresis [16,17]. Therefore, a general prophylactic central lymph node resection for pT1-pT2 papillary tumors is not, and a risk factor-adapted approach is recommended in the ESMO guidelines [18]. However, on papillary carcinoma >pT1a, the German guidelines recommend a prophylactic central lymphadenectomy, depending on the surgeon´s expertise [13]. It remains to be seen to what extent this recommendation will endure, as the guideline is expected to be updated in 2024. However, since our analysis included a central lymphadenectomy in the thyroid cancer group, we could not differentiate whether the risk of paresis was due to the tumor characteristics or the lymphadenectomy.

Although a higher risk for RLN palsy in extended surgeries (total thyroidectomies) compared to subtotal resections has been reported [19,20,21], our study presents a higher risk for RLN palsy in hemithyroidectomies than in total thyroidectomies (11% RLN palsy in hemithyroidectomies and 6% RLN palsy in total thyroidectomies). However, it should be noted here that in cases of aborted total thyroidectomy due to LOS for the first side, the patients were assigned to the hemithyroidectomy group. This could be an explanation for the fact that hemithyroidectomy was associated with a higher risk of RLN palsy than total thyroidectomy and that in total thyroidectomy, Graves’ disease missed the significance level as a risk factor, but, in hemithyroidectomy, it had a 14-fold increased risk for RLN palsy compared to benign nodular goiter.

In our study, less experienced surgeons performed 28% of the hemithyroidectomies under supervision, in contrast to total thyroidectomies with 19%. On the other hand, it has been shown that supervised trainees can perform thyroid surgery for even Graves’ disease safely if a standardized surgical teaching program is available [22]. However, with an overall rate of 6.1% for transient and 1% for permanent RLN palsy, our complication rate is in line with the literature [2].

We could not find any significant interaction of sex, thyroid size, BMI, and RLN palsy, either in thyroidectomy or hemithyroidectomy. High BMI was associated with longer operation periods but not with increases in RLN palsy [23,24]. On the other hand, our results indicate a correlation between longer operation duration and RLN palsy. The duration of surgery per se depends on many factors such as the operation type, underlying diagnosis, experience of the surgeon, and patient-dependent factors. An extended duration of the operation alone can be associated with a longer period of intralaryngeal compression and traction of the RLN, resulting in neuropraxia [25]. It is postulated that a longer operation duration alone correlates with an increased difficulty level of the operation. BMI, male gender, and the presence of peri thyroid adhesions were identified as potential factors, and a probability score was defined [26]. However, the role of gender and BMI with regard to RLN palsy was inconclusive [10,25]. Male sex might be associated with higher RLN palsy, but, in our study, no gender- or BMI-specific differences were revealed.

Some data have demonstrated a higher risk for RLN palsy in thyroidectomy for the right side, which might be due to the smaller RLN diameter [27]. Therefore, stretching sensations could have a more deleterious effect on the right side. Even in a porcine model, the higher stiffness of the left RLN was associated with a lower risk of palsy [28]. In our study, no significant differences between left- and right-side RLN palsy could be detected, so the side alone was not a risk factor for palsy per se.

Taken together, surgery duration and diagnosis (Graves’ disease, thyroid cancer, and recurrent goiter) are significantly associated with a higher risk of RLN palsy, with high specificity but low sensitivity. Especially for the diagnoses mentioned above, a more experienced surgeon should perform the operation. Certainly, the retrospective study design presents limitations, and studies with a bigger data pool, i.e., register studies, are more conclusive. The strength of our data is that in all operations, intermittent neuromonitoring was used, ensuring an adequate comparison of the groups.

## Figures and Tables

**Table 1:** Patient characteristics subdivided into all patients, patients with RLN palsy, and patients with no RLN palsy.

Characteristics	Total (N = 1147)	No RLN Palsy Thyroidectomy (THY) (N = 471)	RLN Palsy THY (N = 30)	No RLN Palsy Hemithyroidectomy (HEMI) (N = 575)	RLN Palsy HEMI (N = 71)
Gender ^1^					
Female	854 (74.5)	361 (76.6)	25 (83.3)	413 (71.8)	55 (77.5)
Male	293 (25.5)	110 (23.4)	5 (16.7)	162 (28.2)	16 (22.5)
Surgery ^1^					
Total Thyroidectomy	501 (43.7)	471 (100)	30 (100)	-	-
Hemithyroidectomy	646 (56.3)	-	-	575 (100)	71 (100)
Indication ^1^					
Graves’ Disease	180 (15.7)	154 (32.7)	10 (33.3)	7 (1.2)	9 (12.7)
Thyroid Cancer	125 (10.9)	52 (11)	5 (16.7)	56 (9.7)	12 (16.9)
Recurrent Goiter	26 (2.3)	10 (2.1)	1 (3.3)	10 (1.7)	5 (7)
Benign Nodular Goiter	810 (70.6)	254 (53.9)	13 (43.3)	498 (86.6)	45 (63.4)
Others	6 (0.5)	1 (0.2)	1 (3.3)	4 (0.7)	-
Nerves at risk (side) ^1^					
Left	843 (51.2)	471	30	301 (52.3)	41 (57.7)
Right	805 (50.8)	471	30	274 (47.7)	30 (42.3)
RLN palsy/nerve at risk (n = 1648) ^1^					
Left	54 (3.3)	-	13	-	41
Right	47 (2.9)	-	17	-	30
No palsy					
Total palsy	101 (6.1)				
Surgeon experience ^1^					
Experienced	870 (75.9)	380 (80.7)	24 (80)	418 (72.7)	48 (67.6)
Non-experienced	277 (24.1)	91 (19.3)	6 (20)	157 (27.3)	23 (32.4)
Re-Surgery ^1^					
Yes	94 (8.2)	24 (5.1)	4 (13.3)	59 (10.3)	7 (54.6)
No	1053 (91.8)	447 (94.9)	26 (86.7)	516 (89.7)	64 (45.4)
Age ^2^	51.9 (13.98)	52.8 (14.2)	47.9 (17.32)	51.6 (13.5)	50.4 (14.74)
BMI ^2^	28.8 (6.02)	28.5 (6.09)	27.7 (6.81)	28.9 (5.85)	29.5 (6.58)
Surgery Duration (Min) ^2^	72 (30.12)	84 (31.51)	99 (35.05)	60 (22.73)	72 (30.72)
Duration of Stay (D) ^2^	5 (2.2)	5 (1.7)	6 (2.79)	5 (1.3)	6 (6.2)
TSH ^2^	1.3 (2.8)	1.22 (2.95)	1.61 (2.22)	1.32 (2.54)	1.54 (3.93)
T3 ^2^	3.36 (1.32)	3.57 (1.87)	3.26 (1.4)	3.18 (0.5)	3.45 (1.19)
T4 ^2^	1.25 (0.7)	1.28 (0.75)	1.11 (0.27)	1.24 (0.72)	1.19 (0.31)
Post-Operative Thyroid Weight ^2^	55.7 (60.8)	74.9 (72.2)	105.2 (124.98)	38.1 (33.8)	50.2 (60.45)

^1^ Frequencies are expressed with percentages. ^2^ Means are expressed with standard deviations.

**Table 2:** Logistic regression with the individual variables.

	Odds Ratio	95% CI ^1^		DF ^2^	*p* ^3^
		Lower Bound	Upper Bound		
Graves’ disease vs. other indications	1.622	0.942	2.795	1	0.133
Thyroid cancer vs. other indications	2.164	1.217	3.848	1	0.015
Recurrent goiter vs. other indications	4.124	1.595	10.661	1	0.003
BMI	1.014	0.981	1.048	1	0.41
Gender (female vs. male)	1.339	0.812	2.207	1	0.242
Surgery duration	1.009	1.003	1.015	1	0.005
Surgery method (hemithyroidectomy vs. total thyroidectomy)	1.939	1.244	3.022	1	0.003
Resection weight	1.003	1.000	1.005	1	0.086
Surgeon experience (experienced vs. inexperienced)	0.772	0.490	1.215	1	0.270

^1^ 95% confidence interval. ^2^ Degrees of freedom. ^3^ *p*-value.

**Table 3:** Logistic regression only with the total thyroidectomies.

	Odds Ratio	95% CI ^1^		Df ^2^	*p* ^3^
		Lower Bound	Upper Bound		
Graves’ disease vs. other indications	1.183	0.513	2.728	1	0.694
Thyroid cancer vs. other indications	1.751	0.604	5.074	1	0.296
Recurrent goiter vs. other indications	1.821	0.218	15.249	1	0.575
BMI	1.007	0.949	1.068	1	0.823
Gender (female vs. male)	1.514	0.566	4.049	1	0.390
Surgery duration	1.012	1.003	1.022	1	0.019
Resection weight	1.004	1.000	1.007	1	0.065
Surgeon experience (experienced vs. inexperienced)	0.958	0.380	2.412	1	0.928

^1^ 95% confidence interval. ^2^ Degrees of freedom. ^3^ *p*-value.

**Table 4:** Logistic regression only with the hemithyroidectomies.

	Odds Ratio	95% CI ^1^		Df ^2^	*p* ^3^
		Lower Bound	Upper Bound		
Graves’ disease vs. other indications	14.343	5.101	40.326	1	<0.001
Thyroid cancer vs. other indications	2.390	1.194	4.785	1	0.012
Recurrent goiter vs. other indications	5.578	1.827	17.027	1	<0.001
BMI	1.015	0.975	1.057	1	0.465
Gender (female vs. male)	1.358	0.305	2.439	1	0.295
Surgery duration	1.017	1.008	1.027	1	<0.001
Resection weight	1.006	1.001	1.012	1	0.023
Left and right nerve at risk (right nerve vs. left nerve)	0.796	0.483	1.310	1	0.367
Surgeon experience (experienced vs. inexperienced)	0.784	0.461	1.331	1	0.373

^1^ 95% confidence interval. ^2^ Degrees of freedom. ^3^ *p*-value.

**Table 5:** Multiple logistic regression of significant variables.

	Odds Ratio	95% Confidence Interval		Df ^1^	*p* ^2^
		Lower Bound	Upper Bound		
Surgery duration	1.015	1.008	1.021	1	<0.001
Surgery indication (Graves’ disease, thyroid cancer, recurrent goiter vs. benign nodular goiter, other indications)	2.631	1.660	4.169	1	<0.001
Surgery method (hemithyroidectomy vs. total thyroidectomy)	3.989	2.364	6.731	1	<0.001

^1^ Degrees of freedom. ^2^ *p*-value.

## Data Availability

All data generated or analyzed during this study are included in this published article.
